# Supplementary material for: Are carbon emissions trading and green financial instruments synergistic? -Comprehensive quantitative research based on content analysis
Source: PLoS One. 2024 Mar 7;19(3):e0298601. doi: 10.1371/journal.pone.0298601 (PMC10919601; doi:10.1371/journal.pone.0298601)
Supplement: S1 Data — (ZIP) [file pone.0298601.s001.zip › Data/地级市Z_F工作报告.zip!/┴╔─■/░░╔╜╩╨/└┤╫╘╣1⁄2╓┌║┼í╛┬φ┐╦╩2╛▌═°í┐_2018.docx]

各位代表：

现在，我代表市政府向大会报告工作，请予审议，并请市政协各位委员提出意见和建议。

一、过去五年工作回顾

过去五年，在鞍山发展进程中非同寻常。五年来，我们在省委、省政府和市委的正确领导下，以习近平新时代中国特色社会主义思想为指引，持之以恒落实新发展理念和“四个着力”“三个推进”，面对净化修复政治生态的艰巨任务，面对经济下行的巨大压力，面对历史遗留的沉重包袱，我们始终坚定信心，保持定力，攻坚克难，战胜了诸多挑战，经受了种种考验，付出了艰苦努力，全市呈现经济平稳发展、民生持续改善、社会和谐稳定的良好局面，为全面振兴钢都打下了坚实基础。

——改革开放不断深化。五年来，我们把改革作为破解难题的金钥匙，坚持全面、系统、协调推进各项改革。“放管服”改革持续深化，行政权力精简了74.2%。市场活力进一步显现，新增各类市场主体6.7万户。成立营商环境办公室和行政审批局，集中开展专项整治，营商环境明显改善。国有企业改革取得重要进展，新组建11家市属国有企业集团，资产总额2320亿元，竞争力进一步提升。民营经济实力增强，占全市经济比重提高到68%。对外开放取得明显成效，跨境电商实现“零”的突破，出口额增长12.5%，进口额增长22.1%。

——产业结构持续优化。五年来，我们立足长远发展，坚定不移调结构。钢铁产业减量提质，淘汰一批落后产能，深加工能力提升30%。下定决心整治菱镁产业“乱象”，“小散低乱”局面有效遏制，菱镁深加工率提高20%。装备制造业向高端化、特色化迈进，信息化与工业化融合发展局面初步显现。传统服务业转型加快，服务业占地区生产总值比重提高12.5个百分点。农业综合生产能力持续提升，粮食连年丰收，粮食安全有效保证。

——创新能力不断提高。五年来，我们把创新作为振兴发展的第一动力，精准发力，持续用力。全社会研发经费增长26.4%，新增国家级企业研发机构3家，设立院士工作站18个。高新技术企业增加105户，产品增加值增长19.2%。直流输配电、大功率变频、金融物联网等技术填补国内空白。12个项目获国家科技进步奖，成为全国科技进步先进市五连冠城市。

——生态环境持续改善。五年来，我们牢固树立和践行绿水青山就是金山银山的理念，强力推进蓝天、碧水、青山、净土四大工程。高质量办理“治理大气污染”和“加强水资源保护”议案。完成污染减排项目768个，万元GDP能耗下降10%。近四年市区环境空气质量达标率提升14.6个百分点。污水处理率由30%提高到95%以上。矿山生态治理面积3.27万亩，完成荒山造林面积23万亩。

——民生改善步伐加快。五年来，我们始终把保障和改善民生放在首位，坚决防止经济下行压力传导到民生。民生投入占公共财政支出比重超过80%。城乡居民人均可支配收入年均增长7.5%和9.3%，城乡低保标准年均提高10%和15%。每年新增就业人数超过4万人，获评全国就业工作先进市。脱贫攻坚战取得重大进展，55个贫困村销号、5.2万名贫困人口脱贫。教育事业均衡发展，医疗卫生服务体系更加完善，公共文化服务水平不断提升，全民健身活动广泛开展，圆满完成“六五”普法工作，六次蝉联“全国双拥模范城”，各项社会事业全面进步。

——历史难题有效破解。五年来，我们不回避、不埋怨、不推脱，勇于担当，尽心竭力解决历史遗留问题。累计投入375亿元，改造棚户区12.5万套，建成回迁房441万平方米，在外漂泊多年的4.8万户居民喜迁新居。为鞍钢办理58平方公里土地证，增加资产208.5亿元，解决了困扰鞍钢十余年的历史难题。应急接管原北美公司供热行为，投入9.5亿元实施并网改造，解决了17.5万户居民反映多年的供暖质量差问题。实施全日制供水改造工程，结束了市区5万多户居民定时供水的历史。依法解决搁置七年的高新广场项目问题。筹措资金3.4亿元，停工七年的公共卫生中心终于建成投入使用。累计化解历史信访积案3000余件，信访总量下降85%。同时，我们坚持实事求是的思想路线，挤掉了多年来形成的水分，做实了经济数据，为全市经济健康发展打下了良好基础。

各位代表，刚刚过去的2017年，我们坚持把习近平新时代中国特色社会主义思想作为破解鞍山改革发展问题的根本遵循，全面落实新发展理念和“四个着力”“三个推进”，实施了全面振兴钢都全链条工作举措，形成了解决振兴发展难题的鞍山方案，全市经济社会发展呈现崭新局面。预计，全年实现地区生产总值1597.5亿元，增长6.5%；规模以上工业增加值334.5亿元，增长7%；一般公共预算收入140亿元，增长5.3%；城乡居民收入均增长6.5%。

（一）坚持稳中求进，经济运行企稳向好

全力稳定经济增长。加强经济运行调度，坚持“一企一策”精准帮扶，领导干部包保重点企业，解决各类难题241个。组织银企对接30余次，推介项目328个。帮助12户企业化解了29.3亿元贷款逾期风险。设立“市长企业家接待日”，帮助56户企业解决生产经营难题119个。深入开发园区现场办公74次，实地解决各类问题200多个。新增规模以上工业企业86户。规模以上工业利润增长2倍。

全力扩大有效投资。加大招商引资力度，洽谈推进亿元以上项目245个，签约99个。开工建设亿元以上项目395个，竣工投产45个。加强与鞍钢合作，建设6大产业园区，推进28个重点项目，煤焦油深加工等一批重点项目开工建设。

全力促进消费、扩大出口。大力发展电子商务和现代物流业，亚马逊跨境电商平台落户鞍山，新增限额以上企业150户，电商交易额突破400亿元，会展交易额超过90亿元。积极融入“一带一路”建设，西柳海关监管场所主体竣工，打通“蒙俄欧”和“新亚欧”国际物流大通道。全年出口135亿元，增长18.4%，创历史最好水平。

（二）坚持改革开路，发展活力不断释放

进一步优化营商环境。新取消市级行政权力82项，下放122项。做实行政审批局，系统化再造审批流程，审批环节压缩36.6%，层级压缩25.1%，办理时限压缩15.3%。全面实行“多证合一、一照一码”。在市县两级全面推开企业投资项目承诺制。全年新增各类市场主体3.98万户，增长38.9%。

深化行政体制改革。推进县（市）区管理体制改革，累计下放服务事项1216项。精简开发园区数量，整合内部机构，强化招商服务功能，园区发展活力进一步增强。扎实推进事业单位分类改革，核减事业单位编制8823个。在全省率先实行公安“大部制、大警种”改革，深化城市管理综合行政执法体制改革，行政效能大幅提升。

深化国资国企改革。新组建市温泉集团、矿业集团、菱镁集团。市燃气集团混合所有制改革迈出重大步伐。全市国有企业引进投资35.8亿元。与驻鞍央企签订“三供一业”分离移交协议，移交费用33.4亿元。

（三）坚持创新驱动，发展动能不断集聚

新兴产业加快发展。九次方大数据公司落户鞍山，梦网云数据计算中心建成。九夷锂电项目即将投产，新能源汽车项目深入推进。“中国磁谷”规划完成。千山风景区晋升国家5A级旅游景区，全市旅游业总收入增长13%。

新业态不断涌现。建设“创谷”5家，引进城市合伙人5个。设立引导资金2000万元，培育科技型企业518户，其中规模以上企业157户，主营业务收入增长9.5%，利润总额增长325.4%。

新技术加速转化。68户企业与中科院等知名院所实现对接。辽宁科技大学与地方合作实施50个重点项目。建成鞍山科技大市场网上技术交易中心。完成科技成果转化65项，16个项目获得省科学技术奖，增长54%。

创新环境初步形成。设立40亿元股权投资母基金，首期10亿元规模。新增基金管理公司5家，组建各类基金6支。鞍山科技金融超市投入运营。建成人才产业园，设立人才发展专项资金3亿元，引进院士4名、各类专家120余人。高新区成为国家“双创”示范基地。

（四）坚持质量引领，产业竞争力不断提升

全面开展质量提升行动。加速推进质量、标准、品牌、信誉“四位一体”建设。聚龙股份、福鞍集团分别获省长质量金奖和银奖。发布实施国家级标准6项。森远路桥等17个产品成为辽宁名牌产品，鞍山南果梨入选中国百强农产品区域公用品牌。质量竞争力指数达到79.3%，创历史新高。

扎实推进“三去一降一补”。淘汰34户“地条钢”企业落后产能460万吨。商品房销售面积增长12%，住宅去化周期减少10个月。争取地方政府性债务置换债券195亿元，全市节约债务利息成本5亿元。落实行政事业性收费优惠政策，减轻企业负担9400万元。帮助71户企业获得直接交易电量34.6亿千瓦时，降低企业成本7761万元。

着力调整优化产业结构。加快钢铁、菱镁、装备制造产业创新发展，推进高炉减量置换，整合菱镁矿产资源开发，加速推进轻型燃气轮机等先进装备制造业项目，三大传统产业实现利润增长2.3倍。新增金融机构6户，在新三板挂牌企业2户，全市金融业完成税收17.9亿元；德邻陆港、龙基物流园等项目加速建设。调减玉米种植面积8.4万亩，新增绿色食品基地5000亩；海城农村改革试点稳步推进，形成一批可复制经验；完成国有林场改革。

（五）坚持为民利民惠民，切实保障改善民生

成功跨入全国文明城市行列。全力开展创城攻坚，圆了鞍山18年的“创城梦”。维修改造道路46.3万平方米，改造老旧小区214万平方米，新建休闲街心游园14个，新增城市绿地51.5万平方米。完成棚户区改造18778套。二一九公园改造全面完成，玉佛山新铺设环保道路3.3公里。开通鞍山至南京航线，鞍山机场改扩建项目加快建设。沈海高速汤岗子出口建成通车，鞍山北出口连接线开工建设。完成智慧城市顶层设计，建成市政府大数据中心，稳步推进城市网格化建设。

深入实施精准扶贫、精准脱贫。投入扶贫资金3.1亿元，完成道路硬化251.7公里，改造农村危房3171户，实施项目扶贫2301户，资助贫困家庭学生1317名，帮助贫困户解决就业1591人，实现27个贫困村、8455人脱贫。妥善应对岫岩特大暴雨灾害，提前转移群众10万多人，人民群众的生命财产损失降到了最低；举全市之力开展灾后重建，投入2.6亿元，救助受灾群众1.2万人，重建倒损房屋1476户，农村饮水、道路、电力通讯等基础设施恢复重建全面完成，工农业生产全面恢复。

积极推进生态环境治理。中央环保督察组交办的500个信访案件，整改完成426件。投资21亿元，完成大鞍山生态区建设项目26个。取缔10吨以下燃煤锅炉499台，淘汰黄标车及老旧车辆11680辆。市区环境空气质量达标率72.1%。完成3座污水处理厂提标改造和灵山明沟等5条黑臭水体治理。

各项社会事业健康发展。为城乡群众办的20件实事扎实推进。全市新增就业4.5万人，登记失业率控制在2.98%。推进机关养老保险改革。弥补企业养老保险资金缺口54亿元，40万企业离退休职工养老金及时足额发放。新增学前教育学位8000个，完成农村薄弱学校改造110所。积极推进国家职业教育“双元”培育试点工作。加快分级诊疗制度建设。开通全国跨省地级就医直接结算。开展送戏下乡130场。新增健身器材2150件。市新殡仪馆建成使用。市科技馆、档案馆主体竣工，市青少年活动中心、妇女儿童活动中心加快建设。社会治安综合治理取得明显成效，圆满完成十九大维稳安保任务，信访工作取得历史最好成绩，全年未发生重特大安全事故和食品药品安全事件，全市社会大局保持和谐稳定。广播电视、新闻出版、老龄、残联、民族、宗教、外事、侨务、对台、统计、接待、档案、编制、气象、地震、民兵预备役等工作取得新成绩。

（六）坚持全面从严治党，不断加强政府自身建设

牢固树立“四个意识”，始终坚持一手抓振兴发展，一手抓全面从严治党。深入推进“两学一做”学习教育常态化制度化，加强党风廉政建设，切实履行“一岗双责”，坚决落实中央八项规定，严厉整治各种顶风违纪行为。坚持依法行政，建设法治政府，认真办理人大代表建议、政协提案487件，见面率、办复率均为100%。切实转变工作作风，大力弘扬实干精神，不做表面文章，不喊空洞口号，对于每一项重点工作都确定时间表、制定路线图、明确责任人，全市上下呈现出齐心协力共谋发展，凝心聚力干事创业的浓厚氛围。

各位代表，经过五年的不懈奋斗，鞍山发生了令人欣喜的变化，取得了令人鼓舞的成绩。这些成绩的取得是习近平新时代中国特色社会主义思想正确指引的结果，是省委、省政府和市委坚强领导的结果，是市人大、市政协有效监督和鼎力支持的结果，是全市人民共同奋斗的结果。在此，我代表市政府向全市广大干部群众，向各民主党派、各人民团体和各中省直单位，向关心支持鞍山发展的海内外朋友，表示衷心的感谢和崇高的敬意！

五年的工作实践，我们深刻体会到，做好政府工作，必须旗帜鲜明讲政治，坚决维护以习近平同志为核心的党中央权威和集中统一领导，坚决贯彻习近平新时代中国特色社会主义思想和“四个着力”“三个推进”，坚决贯彻省委、省政府和市委的决策部署。必须坚持以人民为中心的发展思想，把增进人民福祉、增强群众获得感作为最大的追求；必须坚持高质量发展,用新理念引领发展，以改革破解难题，以创新提升动力，提高发展的质量和效益；必须始终坚持求真务实、真抓实干，以扎扎实实的工作作风，追求实实在在的发展成效。

在总结成绩的同时，我们也清醒地看到，鞍山经济社会发展中还存在不少短板，发展不平衡不充分的问题比较突出。一是经济下行压力依然较大，部分实体经济经营困难，重大项目、新开工项目和竣工达产项目较少。二是产业转型升级任务较重，“一业独大”的现状没有根本性转变，新业态、新模式不足，创新能力不强，新产业尚未形成有效支撑。三是营商环境建设任重道远，部分干部不愿作为、不敢作为、不会作为，服务意识、服务能力、服务水平需要进一步提高。四是民生领域仍有不少短板，生态环境欠账较多，脱贫攻坚任务较重，就业、教育、医疗、养老等方面还面临不少难题。五是防范和化解风险任务依然艰巨，政府性债务负担过重，企业养老金缺口较大，部分企业负债率较高。这些问题我们将在今后的工作中采取有效措施，认真加以解决。

二、今后五年的奋斗目标和主要任务

今后五年是全面建成小康社会的攻坚期，是全面振兴钢都的关键期，是实现高质量发展的机遇期。鞍山发展进入新时代，全面振兴钢都开启新征程。我们必须不忘初心、牢记使命，以永不懈怠的精神状态和一往无前的奋斗姿态，走出一条新理念指导下的振兴钢都之路。

今后五年的指导思想是：高举中国特色社会主义伟大旗帜，全面贯彻落实党的十九大精神，以习近平新时代中国特色社会主义思想为指导，坚持以人民为中心，坚持稳中求进工作总基调，坚持新发展理念，按照高质量发展的要求，统筹推进“五位一体”总体布局和协调推进“四个全面”战略布局，深入推进供给侧结构性改革，深度融入“一带五基地”建设和“五大区域发展战略”，聚焦“一条主线、三大任务、四个目标”，落实“四个着力”“三个推进”全面振兴钢都全链条部署，决胜全面建成高质量小康社会，谱写鞍山老工业基地全面振兴的新篇章。

今后五年的发展目标是：在2020年全面建成高质量小康社会的基础上，到2022年全面深化改革取得决定性进展，现代化经济体系基本建立；创新能力进一步增强，新动能成为发展的重要引擎；发展质量效益进一步提升，产业竞争力进一步增强；转型升级取得重大突破，产业结构更加科学；经济保持中高速增长，产业迈向中高端水平；城市品质更加彰显，生态环境更加宜居宜业；人民生活更加殷实，人民群众获得感、幸福感、安全感更加充实、更有保障、更可持续，为2030年实现全面振兴钢都宏伟目标，2035年基本实现社会主义现代化、再创钢都辉煌奠定坚实基础。

今后五年的主要任务是：

——深化改革开放,打造更具要素集聚和辐射力的区域城市，进一步提升鞍山城市影响力。重点领域关键环节改革取得新突破，经济环境、政务环境、市场环境、营商环境、开放环境进一步优化。紧紧抓住“一带一路”战略机遇，借鉴辽宁自贸区经验，以园区为平台，建设开放高地；以龙头企业为引领，组建联盟“抱团出海”；以引进人才和项目为抓手，增强发展后劲；以优化营商环境为保障，打造发展环境最优城市。实现出口额年均增长5%以上，进口额年均增长10%以上，在全省“一带五基地”建设中勇立潮头，在沈阳经济区一体化发展中走在前列。

——推进结构调整，打造更高水平的产业创新示范城市，进一步提升鞍山产业竞争力。供给侧结构性改革取得阶段性成果，过剩产能有效化解，发展质量效益明显提高，基本建成战略性新兴产业和传统制造业并驾齐驱、现代服务业和传统服务业相互促进、信息化和工业化深度融合的现代化产业发展新格局。打造钢铁、菱镁2个千亿级产业集群，培育先进装备制造、精细化工、钢铁物流、新一代信息技术、新能源汽车、磁应用6个百亿级产业集群。传统商贸业转型升级取得新突破，健康旅游等现代服务业加速发展。实施乡村振兴战略成效显著，县域经济快速崛起。第一产业增加值年均增长3%以上，工业增加值年均增长8%左右，社会消费品零售额年均增速高于全省平均水平。

——培育发展新动能，打造更宜创新创业的活力城市，推动鞍山实现高质量发展。建立创新创业生态系统，推进数字经济、平台经济、共享经济、智慧经济等新经济业态快速发展。建立以企业为主体、市场为导向、产学研深度融合的创新体系，高新技术企业超过350户。全社会研发经费占地区生产总值比重达到2.5%以上，科技成果转化项目300个以上。加快高新区“双创”示范基地建设，力争进入国家级高新区50强；经开区打造成为国家级开发区。全社会创新创造活力竞相迸发，大众创业、万众创新成为引领经济增长的新引擎、转型升级的新动能，跻身国家创新型城市。

——增进民生福祉，打造更高质量的幸福城市，使人民在共建共享发展中有更多获得感。始终坚持以人民为中心的发展思想。办好人民满意的教育，打造省内一流教育强市，关键指标进入全国先进行列。城乡居民就业质量不断提升，高水平社会保障体系初步建立，完成城乡居民和各类群体收入增长三年行动计划，坚持在经济增长的同时实现居民收入同步增长、在劳动生产率提高的同时实现劳动报酬同步提高。确保到2020年现行标准下农村贫困人口实现脱贫，岫岩县摘掉贫困县帽子。加快“健康鞍山”建设，打造区域医疗中心城市。持续巩固文明城市成果，提升城市品质，基本建成智慧城市。加快大鞍山生态区建设，城乡生态环境明显改善。深入实施文化惠民工程，建设体育强市。支持国防和军队建设，推进军民融合深度发展，继续创建“全国双拥模范城”。促进社会公平正义，形成有效的社会治理和良好的社会秩序。

三、2018年主要工作

2018年是贯彻党的十九大精神的开局之年，是改革开放40周年，是决胜全面建成小康社会、实施“十三五”规划承上启下的关键一年，是落实全链条部署、走实走好全面振兴钢都新路的重要一年。我们将在省委、省政府和市委的正确领导下，按照高质量发展的要求，坚持稳中求进工作总基调，以供给侧结构性改革为主线，大力推进改革开放，深入实施创新驱动，推动地企深度融合，打好三大攻坚战，加强和改善民生，促进经济社会持续健康发展。力争实现地区生产总值增长7%，固定资产投资增长15%，社会消费品零售额增长8%，一般公共预算收入增长7%，规模以上工业增加值增长7%，城镇登记失业率控制在3.5%以内，城乡居民收入增长与经济发展同步。

在新的一年里，着力做好以下工作：

（一）坚定不移推进供给侧结构性改革

把改造提升传统优势产业作为稳增长的压舱石，把培育壮大新兴产业作为推进动能转换的加速器，加快建设现代化产业体系，促进高质量发展。

支持鞍钢做强做优做大。发挥鞍钢在供给侧结构性改革中的龙头带动作用。举全市之力支持鞍钢发展，促进鞍钢实施“631”结构调整规划。全力支持鞍钢深化改革、承接好“三供一业”分离移交，支持厂办大集体改革，帮助鞍钢卸下包袱、轻装上阵，助推鞍钢成为具有较强创新能力和国际竞争力的钢铁企业集团。深化地企融合发展，充分发挥鞍钢资本优势和地方要素优势，全面开工建设地企共建6大产业园区。成立驻鞍央企工作办公室，支持中省直企业在鞍山加快发展。

加速推进产业转型升级。提升钢铁精深加工水平，打造菱镁深加工企业集团，深入实施装备制造三年发展计划，发展节能环保产业，建设军民融合产业园。培育壮大新兴产业。加快发展新一代信息技术产业，推进大数据产业基地建设；大力发展磁应用产业，建设“一镇两园三中心六大基地”；做大新能源汽车产业，建设中德产业园鞍山分园和新能源电池产业园；大力发展健康旅游产业，建设一批精品旅游项目，办好中国工业遗产年会，实现旅游总收入增长13%。促进新动能持续快速成长。积极融入沈大国家自主创新示范区，发展壮大“金融+”，全力推进“哈工大—智谷”等创谷建设，培育科技小巨人企业20户以上，引进一批城市合伙人。加速推进辽宁科技大学与地方合作重点项目。全年新增科技成果转化60项以上。大力发展现代服务业。加快万达广场等一批项目建设，推进新零售等多业态叠加、智慧服务、线上线下融合发展。

深入推进“三去一降一补”。进一步淘汰落后产能，减少低端无效供给，为经济发展腾出空间。着力推动房地产去库存，出台优惠政策，促进商品房销售，合理缩短去化周期。全面落实减税降费政策，清理涉企收费，降低制度性交易成本。

（二）凝神聚力实施项目建设攻坚行动

把项目建设作为促进经济发展的第一要务，把招商引资作为“一号工程”，实施项目建设三年攻坚行动计划，彻底扭转项目建设被动局面。

加大项目谋划力度。按照新发展理念谋划项目，把高质量的项目建设作为推进高质量发展的重要支撑。围绕落实新一轮东北振兴等一系列国家政策，聚焦先进装备制造、现代服务业、现代农业、基础设施、社会事业、生态环保六大领域，突出新一代信息技术、新能源汽车、磁应用、精细化工、健康旅游、现代物流六大主攻方向，谋划一批大项目、好项目。

加快项目落实落地。将项目建设作为领导干部绩效考核的重要内容,严格落实责任，制定奖励办法，兑现优惠政策。坚持全员招商、以商招商、亲情招商、诚信招商、务实招商。建立投资项目代办帮办制度和联席会议制度，一个项目，一个领导、一个团队，一抓到底，帮助企业解决项目推进中遇到的征地、拆迁、资金等方面的困难和问题。坚持引资与引智并举，引才与荐才并重，办好人才产业园，吸引更多人才来鞍创业。全年力争开工建设重点项目300个以上，其中亿元以上项目力争达到200个以上。

（三）满怀真情促进民营经济加快发展

民营经济是鞍山发展的重要支撑，企业家是钢都振兴的宝贵资源。把加快民营经济发展摆在更加突出的位置，使民营企业成为推动发展、增强活力的重要力量。

营造有利于民营经济发展的浓厚氛围。着力打造安商亲商富商的人文环境，大力弘扬企业家精神，对民营企业多关注、多关心、多引导，真心实意支持企业发展。对作出重要贡献的企业进行嘉奖。全力调动本地企业家发展积极性，视企业家为亲人，用乡土情怀激发本地企业家投资发展热情。诚邀鞍山籍企业家回乡创业、报效家乡、圆梦鞍山。

打造有利于民营经济发展的营商环境。牢固树立营商环境就是生产力的理念，积极构建“亲”“清”新型政商关系，坦坦荡荡、清清白白与企业家交朋友，认认真真、扎扎实实帮助企业解难题。持续推进“放管服”改革，放权更加彻底，管理更加科学，服务更加贴心。推行行政审批“多图联审”“多评合一”“联合验收”三项改革，构建市县乡村四级联动政务服务新体系。着力开放市场准入，放宽民间投资领域。坚决破除政策执行中的“玻璃门”“弹簧门”，当好企业的“店小二”，做到有求必应，无事不扰。

解决民营经济发展中遇到的实际问题。保护民营经济合法权益，着力解决侵害民营企业利益问题，尽最大努力解决一批腾迁净地、拖欠工程款、政策兑现等方面的历史遗留问题。组建民营经济发展智库，帮助企业规划发展前景、谋划战略布局。建设“企业之家”服务平台，宣传解读政策，促进政策落实落地。建立中小微企业服务中心，为中小企业提供一站式、点对点服务。支持企业激活土地、厂房、设备等闲置资产，推进资产证券化，解决企业资产负债率偏高的问题。让鞍山成为民营企业投资兴业的沃土和热土。

（四）积极稳妥推进改革开放形成新格局

把改革开放作为推动发展的“关键一招”，努力把鞍山建设成为“一带一路”的重要节点，成为沈阳经济区的重要支撑。

着力推进重点领域和关键环节改革。加快推进国资国企改革。围绕管资产为主加快转变国有资产监管机构职能，推进市属国有企业集团构建现代企业管理体系，建立灵活高效的市场化经营机制，完成市燃气集团混合所有制改革，积极推进市建投集团引进战略投资者，进一步增强国有企业的整体实力和竞争力。深化财税体制改革。进一步理顺市与县（市）区财权事权关系，积极探索完善县乡财政体制，建立“分灶吃饭、风险共担”的利益分配机制，激发各县（市）区和乡镇经济发展内生动力。深化开发园区体制改革。进一步完善开发园区管理体制、机构设置、用人机制和财政体制，科学规划园区发展空间，探索实行“园中园”管理模式，激发园区活力，促进园区提档升级。推进事业单位分类改革。按照政事分开、事企分开、管办分离的方向，加速推进经营性事业单位转企，适时启动推进我市承担行政职能事业单位改革工作。

发展更高层次的开放型经济。主动对接国家和省区域经济协调发展战略。积极融入“一带一路”，争取西柳国家市场采购贸易试点获批，加快通关平台、跨境贸易平台、物流平台建设，规划建设鞍山港，推进“国检试验区”建设。加快规划、要素、产业、政策全面融合，促进与沈阳经济区一体化发展。积极参与辽宁自贸区、沈大自主创新示范区建设，争取进入首批辽宁自贸试验区协同发展区域。紧紧抓住与南京对口合作机遇，共建现代服务业合作园区。

（五）锲而不舍实施城市品质提升行动

巩固文明城创建成果，切实把绿色发展理念贯彻落实到城市规划建设管理的全过程，进一步建好城市、管好城市，让城市更加美丽和谐，让群众享有更好服务。

推进城市基础设施建设。坚持规划先行，全面系统地完善城市规划。全面推进“城市双修”，提标改造建国大道等9条城市主干路，加快自由东街隧道建设和鞍山机场改扩建，建成沈海高速公路鞍山北出口连接线。规划建设一批城市过街天桥和立体停车场，改造城市老旧小区495万平方米。开工建设玉佛山城市森林公园，提升二一九公园品质。加快推进海绵城市建设，统筹推进给排水、园林绿化、道路、管网等设施建设，提高城市基础设施的承载力。

全面提高城市管理服务水平。实施市政道路、环境卫生和城市绿化的精细化管理，健全完善城市管理常态化长效化机制。深入推进智慧城市建设，建成“三位一体”综合管理指挥平台，积极推进智慧交通、智慧医疗等项目建设。全面整治交通拥堵、马路市场、占道经营、乱停乱放、乱搭乱建、乱贴广告等城市“顽疾”。

进一步改善城市生态环境。抓实抓细大鞍山生态区建设，落实好中央环保督察反馈意见。开工建设生态环保重点项目45项。栽植树木20万株，绿化面积25.8万平方米。淘汰10吨以下燃煤锅炉200台以上。开展运粮河综合整治，扩大污水收集范围，努力实现污水处理厂稳定达标排放。加强矿山排岩场、尾矿库生态修复，强化闭坑矿生态治理管护。开工建设城市污泥处理中心和垃圾处理中心。落实领导干部自然资源资产管理和环境保护责任。

（六）因地制宜实施乡村振兴战略

坚持农业农村优先发展，按照产业兴旺、生态宜居、乡风文明、治理有效、生活富裕的总要求，加快推进农业农村现代化，走中国特色社会主义乡村振兴道路。

推动产业融合发展。深化农业供给侧结构性改革，走质量兴农之路。加快农业转型升级，做强特色农业，做大农产品加工业，做优休闲农业，推动一二三产业融合发展。推进中药材、野生猕猴桃、君子兰等基地建设。推进海城粮食果蔬、台安畜禽、岫岩食用菌等精深加工产业发展，建设亿元以上项目4个、千万元以上项目10个。推进南果梨、君子兰争创中国名牌。推进农业加旅游、康养、文化等多种模式融合，培育市级休闲农业示范村10个。

建设生态宜居乡村。坚持人与自然和谐共生，走乡村绿色发展之路。以美丽乡村、宜居乡村和特色小镇建设为主要抓手，切实改善农村生活环境，构建生态宜居家园。开展垃圾集中治理等四项生态环境治理。实施“厕所革命”等九项基础设施建设。实施生态、美化、文化、产业“四项提升”。高质量推进“四好农村路”建设，新建维修改造农村公路500公里。打造1—2个国家级特色小镇，10个省级特色小镇，建成300个宜居达标村。

夯实农村基层基础。创新乡村治理体系，走乡村善治之路。推广海城农村综合改革试点经验。推进农村土地“三权分置”改革，完成全市农村土地承包经营权确权登记颁证工作，流转土地达到129万亩，流转率达到39%。推进农村集体产权制度改革。加快农垦改革进度。

推进农业经营方式创新。巩固和完善农村基本经营制度，走共同富裕之路。围绕个体农户和现代农业发展有机衔接，构建“土地适度规模经营、新型经营主体、农业社会化服务”模式，带动农户共同发展。培育示范农民合作社10个、百亩以上家庭农场10个。落实好耕地地力保护补贴等各项惠农政策，做好农业保险工作。

（七）全力以赴保障和改善民生

坚持问题导向，倾注真情实感，拿出真招实策，尽力而为，量力而行，扎实办好民生实事，不断满足人民日益增长的美好生活需要。

全力推进精准扶贫、精准脱贫。把提高脱贫质量放在首位，注重扶贫与扶志、扶智相结合，做到扶贫对象精准、项目安排精准、资金使用精准、脱贫措施精准，坚持专项、行业、社会“三位一体”大扶贫格局，实现“两不愁、三保障”，保证农民收入有提高、帮扶成果有提升、集体经济可持续，确保小康路上不落一人。市本级投入扶贫资金6600万元以上，通过光伏电站扶贫、种养殖项目扶贫、乡村旅游扶贫、基础设施扶贫等多种形式，确保年内26个贫困村销号、减贫人口9000人。

加强社会保障体系建设。千方百计筹措资金，确保企业养老金及时足额发放。深化机关事业单位养老保险改革。深化医保支付改革。城乡最低生活保障标准分别提高4%和7%。加大保障房建设力度，大力发展租赁房市场，改造棚户区7000套以上。提高就业质量，全年新增城镇就业4万人。加快养老服务业发展，加强社区养老服务，推广医养结合养老模式。

优先发展教育事业。新建10所公办幼儿园，创建“高幼联盟”，着力解决婴幼儿照护和儿童早期教育服务问题。完成194所薄弱学校改造，实现全域义务教育均衡发展。坚决整治“乱补课”“乱办班”等不良风气，减轻中小学生课外负担。实施市属高中部分教学楼改造工程，推进高中阶段特色化办学。推动鞍山师范学院校区改扩建。成立鞍山市特殊教育学校。推进技术技能人才“双元”培育，建设面向沈阳经济区的职业教育基地。

加快“健康鞍山”建设。深化医药卫生体制改革，在城市建立托管型医联体，在农村建立紧密型医共体。优化公共卫生资源配置，把市中心医院建设成区域医疗中心。扎实推进分级诊疗制度建设，深化家庭医生签约服务，健全乡镇、社区卫生服务体系，提高公共卫生和基本医疗服务水平。

繁荣发展文化体育事业。深入挖掘鞍山传统文化内涵，打造特色文化品牌。实施文化惠民工程，构建公共图书服务、文化活动、公益演出三大配送体系，对接群众需求。引进高端文化演艺公司，支持民营文化场馆建设，提高市民文化消费水平。广泛开展全民健身活动，建成玉佛山体育休闲广场，推进市奥林匹克中心市场化运营。

打造共建共治共享的社会治理格局。多措并举，防范和化解政府性债务、企业养老金支付等风险。健全基层社会治理体系，推进街道和社区体制改革。做好第四次全国经济普查工作。落实“七五”普法责任制。加强平安鞍山建设，依法打击和惩治黄赌毒黑拐骗等违法犯罪活动，着力解决网上虚假信息诈骗、倒卖个人信息等突出问题，创建全国食品安全城市，强化安全生产、信访维稳工作，维护社会大局和谐稳定。

（八）持之以恒加强政府自身建设

把为钢都谋振兴，为百姓谋幸福作为初心和使命，殚精竭虑，夙夜在公，只争朝夕，攻坚克难，着力打造忠诚、为民、法治、务实、廉洁政府。

旗帜鲜明讲政治。牢固树立“四个意识”，坚定“四个自信”，坚决维护以习近平同志为核心的党中央权威和集中统一领导，自觉在思想上政治上行动上同党中央保持高度一致，坚持用习近平新时代中国特色社会主义思想武装头脑、指导实践、推动工作。坚决执行省委、省政府和市委的各项决策部署，坚定不移推进全面振兴钢都，坚定不移推进全面从严治党，坚决惩治腐败，让对党忠诚、为党分忧、为党尽责、为民造福成为政府最鲜明的政治担当。

坚持依法行政。自觉接受人大依法监督和政协民主监督，办理好人大代表建议和政协提案。自觉运用法治思维和法治方式推动工作，坚持法定职责必须为，法无授权不可为。健全科学民主依法决策机制,提高行政决策水平。深化政务公开，让权力在阳光下运行。

坚持真抓实干。新时代千帆竞发、百舸争流，不进则退、慢进亦退。面对振兴发展的历史使命、面对改革创新的艰巨任务、面对人民群众的热切期盼，我们必须以如履薄冰的危机感，逆水行舟的紧迫感，舍我其谁的责任感，咬住目标不放松，靠实干推进钢都振兴，靠实干促进人民幸福，靠实干担当历史使命和时代责任。坚持实字当头，深入基层一线，大兴调研之风，做到决策在一线制定,工作在一线落实,问题在一线解决,成效在一线检验。坚持干字为先，做到任务目标具体化，督查考核常态化，一级做给一级看，一级带着一级干，一件接着一件办，以钉钉子精神推动各项工作取得实效，在新一轮高质量发展中抢得先机、赢得主动。

各位代表，历经风雨的鞍山，已经昂首迈入新时代，阔步踏上新征程。新时代要有新形象，新征程要有新作为。让我们更加紧密地团结在以习近平同志为核心的党中央周围，以习近平新时代中国特色社会主义思想为指导，在省委、省政府和市委的正确领导下，不忘初心，牢记使命，攻坚克难，奋勇争先，为决胜全面建成小康社会，重振钢都雄风，再创钢都辉煌而不懈奋斗！
